# Reusing N95 Respirators at Weekly Intervals During the COVID-19 Pandemic

**DOI:** 10.7759/cureus.13542

**Published:** 2021-02-24

**Authors:** Keitaro Nakamoto, TAKESHI SARAYA, Daisuke Kurai, Naokatsu Fukukawa, Takako Taneoka, Teppei Shimasaki, Haruyuki Ishii

**Affiliations:** 1 Respiratory Medicine, Kyorin University School of Medicine, Mitaka, JPN; 2 General Medicine, Kyorin University School of Medicine, Mitaka, JPN; 3 Infection Control, Kyorin University School of Medicine, Mitaka, JPN

**Keywords:** covid-19, quantitative fit test, n95 respirators, reuse

## Abstract

Objectives

A surge in the demand for N95 filtering facepiece respirators (N95 respirators) due to the worldwide spread of coronavirus disease 2019 (COVID-19) has resulted in a global shortage of N95 respirators. This study was performed to evaluate the clinical validation of reusing N95 respirators following stringent fit test protocols.

Methods

After passing the first fit test, we prospectively enrolled healthcare workers who used N95 respirators for two hours per shift (duckbill-shaped HPR-R/HPR-S, dome-shaped Hi-Luck 350, and three-panel flat-fold respirators 9211) in settings such as bronchoscopy or respiratory specimen sampling. These procedures were repeated for up to three weeks, with the fit test performed every week. At each timing of the fit test, we used a fit-testing system for quantitatively evaluating particle leakage.

Results

A total of 41 participants were enrolled, including 24 doctors and 17 nurses, of whom 25 were women.

The pass rate of successful reuse over three observational weeks using four fit tests was 85.4%, which was comparable among the three types of N95 respirators. Six (14.6%) participants failed the fit test, while no participants dropped out of protocol due to either N95 respirator damage or contamination. Among the six dropped out participants, four reused the duckbill-shaped type and two reused the three-panel flat-fold type. All participants using the cup-shaped type mask successfully completed the protocol. However, the passing rate of this study was not statistically different among the three types of N95 respirators.

Conclusion

This study shows that N95 respirators can be safely reused for a short period irrespective of their type, as quantitatively assessed by fit tests.

## Introduction

Healthcare workers are required to use personal protective equipment to protect themselves from coronavirus disease 2019 (COVID-19) [1]. In particular, the use of N95 filtering facepiece respirators (N95 respirators) is recommended in situations where high-risk aerosol generation is anticipated. However, there is a shortage of N95 respirators in several countries due to the increased demand caused by the COVID-19 pandemic. Therefore, the Centers for Disease Control and Prevention (CDC) suggested reusing N95 respirators [2]. Due to these circumstances, the reuse of N95 respirators was implemented at our hospital. However, the instruction manuals for these N95 respirators do not mention reuse, and there is little evidence as to whether the reuse of N95 respirators is safe. Therefore, we aimed to ascertain whether our hospital’s protocol for reusing N95 respirators is safe for healthcare workers.

## Materials and methods

**Figure 1:**
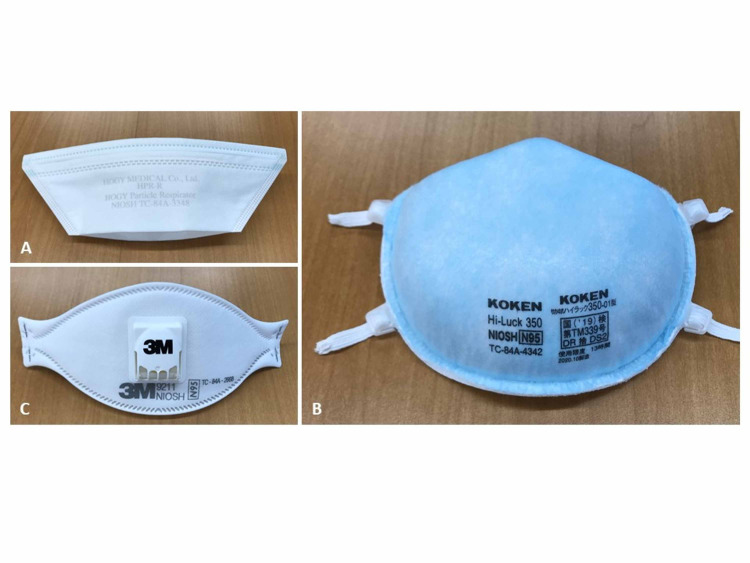
Three types of N95 respirators (A) Duckbill-shaped HPR-R/HPR-S (Hogy Medical Co. Ltd, Tokyo, Japan). (B) Dome-shaped Hi-Luck 350 (Koken Ltd, Tokyo, Japan). (C) Three-panel flat-fold respirators 9211 (3M, St. Paul, Minnesota, MN, USA).

We performed this study at Kyorin University Hospital Tokyo, Japan, from June to July 2020. We prospectively enrolled healthcare workers who used N95 respirators for a short period while at work (e.g., doing bronchoscopy or collecting respiratory samples). Three types of N95 respirators were used, namely, duckbill-shaped HPR-R/HPR-S (Hogy Medical Co. Ltd, Tokyo, Japan) (Figure 1A), dome-shaped Hi-Luck 350 (Koken Ltd, Tokyo, Japan) (Figure 1B), and three-panel flat-fold respirators 9211 (3M, St. Paul, Minnesota, MN, USA) (Figure 1C).

**Figure 2:**
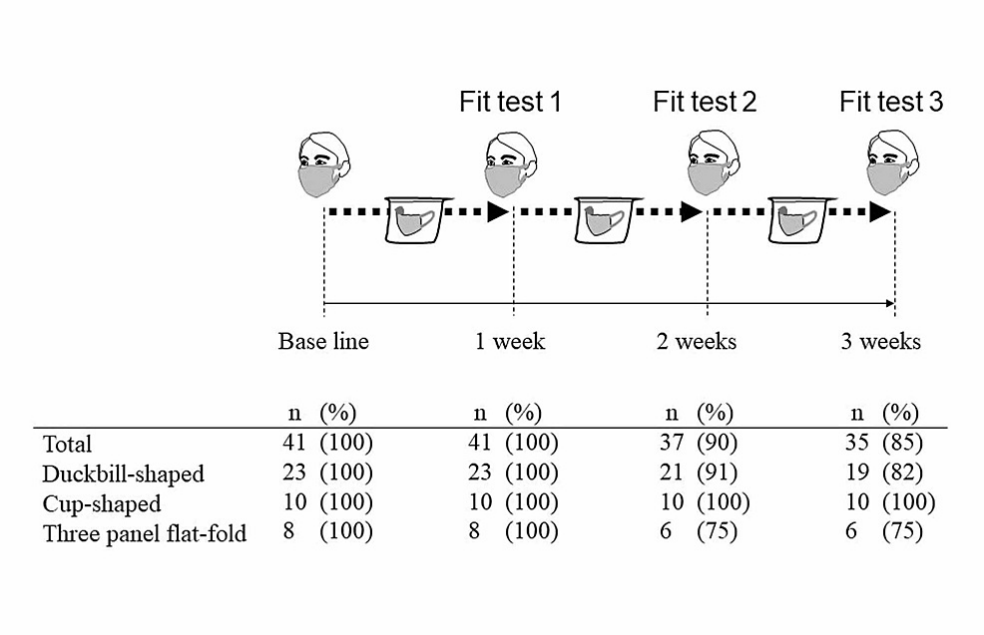
Number of each N95 mask type passed the fitting test Scheme of the protocol used in this study (upper section) and the fit test pass rate results of each type of N95 respirator (lower section).

Our protocol for the reuse of N95 respirators was as shown in Figure 2.

**Figure 3:**
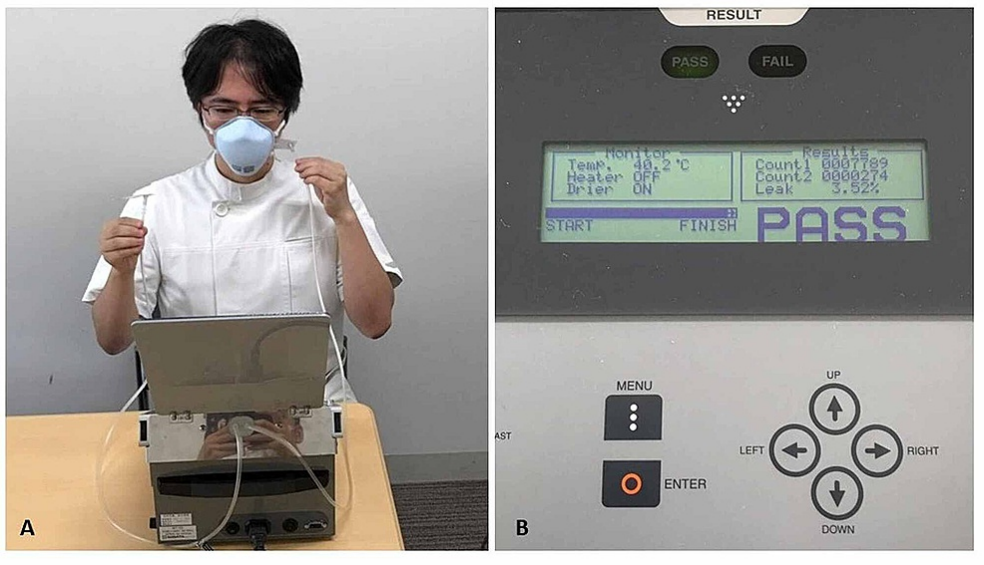
Fit test using the quantitative respiratory fit testing system Physicians insert the test tube into the N95 respirator for keeping them closely attached to the skin (A), which counted the particles in the N95 respirator with relaxed respiration for 30 seconds. Another tube outside of the N95 respirator detected the particles in atmospheric air (A). The leak rate (in this case) was 3.52% (B). The acceptable leak rate for passing the test was 5%.

First, all participants (physicians and nurses) performed a baseline fit test before using an N95 respirator. We used a respirator fit testing system, Model MT-03 (Sibata Scientific Technology Ltd, Saitama, Japan), to evaluate particle leakage while wearing a mask (fit test). Participants inserted the test tube into the N95 respirator to keep them closely attached to the skin (Figure 3A), which counted the particles in the N95 respirator with relaxed respiration for 30 seconds. Another tube was held near the test tube for detecting particles outside the N95 respirator (Figure 3A). Therefore, this device measured the number of particles both inside and outside the respirators.

A pass was defined as a leak rate of less than 5% (Figure 3B).

All participants were required to pass the fit test before using an N95 respirator. After using the N95 respirators, the enrolled participants stored them in a breathable paper bag at the end of the day. N95 respirators were stored without use for one week as the severe acute respiratory syndrome coronavirus 2 (SARS-CoV-2) lives on a surface for a long time [3]. During the storage period, we did not disinfect or sterilize them. The N95 respirators were reused a second time if the participants passed fit test 1 (Figure 2). These procedures were repeated for up to three weeks. At each timing of fit test, if the N95 respirators were apparently damaged or contaminated with blood or body fluids, we discarded them without being reused.

The primary outcome was the pass rate of successful reuse over three observational weeks using four fit tests. The secondary outcome was the pass rate of the three different types of masks. If the participants failed to pass the fit test, they were considered to have dropped out of the protocol and could not proceed to the next step. We recorded the healthcare workers’ experience, type of N95 respirators used, a one-time use per shift, and the fit test pass rates.

Data are presented as the median and interquartile range (IQR) for continuous variables. The number and percentage of participants were considered categorical variables. Differences between groups were assessed using the Kruskal-Wallis test. Categorical variables were assessed using Fisher’s exact test. In all instances, a p-value of <0.05 was considered statistically significant. All statistical analyses were performed using EZR Version 1.35 (Saitama Medical Centre, Jichi Medical University, Saitama, Japan) [4].

This study was approved by the Institutional Review Board and was performed in accordance with the Declaration of Helsinki.

## Results

A total of 41 participants were enrolled, 24 doctors and 17 nurses, of whom 25 were women (Table 1).

**Table 1:** Characteristics of participants Age, years of work experience, and mask use hours/one-time use are presented as median values and interquartile range in parentheses.

	Total (N=41)	Duckbill-shaped type (N=23)	Cup-shaped type (N=10)	Three-panel flat-fold type (N=8)	p-Value
Sex					
Female	25 (100.0)	13 (52.0)	8 (32.0)	4 (16.0)	0.421
Male	16 (100.0)	10 (62.5)	2 (12.5)	4 (25.0)	
Age	36 (32-41)	35 (32-40)	36 (31-39)	39 (31-42)	0.833
Years of work experience	10 (6-16)	10 (6-16)	11 (6-15)	13 (5-17)	0.972
Type of healthcare worker					
Doctor	24 (100.0)	15 (62.5)	3 (12.3)	6 (25.0)	0.134
Nurse	17 (100.0)	8 (47.1)	7 (41.2)	2 (11.8)	
Mask use hours/ one-time use	2.0 (1.7-2.3)	1.9 (1.6-2.2)	2.1 (2.0-2.5)	1.9 (1.6-2.1)	0.131

The different types of N95 respirators used were duckbill-shaped (n=23), cup-shaped respirators (n=10), and three-panel flat-fold respirators (n=8). There was no significant difference in terms of sex or age in each N95 respirator type. There was no dropout case from our protocol due to the N95 respirator damage or contamination.

In total, the median time of reuse per shift was two hours irrespective of the type of N95 respirator used. By mask type, the median time of reuse per shift was 1.9 hours, 2.1 hours, and 1.9 hours for the duckbill-shaped, cup-shaped, and three-panel flat-fold type, respectively. During the observational period, mask use hours per shift were comparable among the three types of N95 respirators (p=0.131).

During the study, 35 (85.4%) participants completed the protocol, but six (14.6%) failed to do so (Figure 2). Among the six failed participants, four reused the duckbill-shaped type and two reused the three-panel flat-fold type. The pass rate of this study was not statistically different among the three types of N95 respirators. Interestingly, all participants passed fit test 1. However, two participants using the duckbill-shaped and three-panel flat-fold type dropped out at fit test 2, and a further two participants using only the duckbill-shaped type dropped out at fit test 3. All participants using the cup-shaped type successfully completed the protocol.

## Discussion

This study demonstrated that 85.4% of participants successfully completed the three-week reuse of N95 respirators protocol using four fit tests taken at weekly intervals. To date, few studies have been conducted on the reuse of N95 respirators. Degesys et al. reported 68 participants who reused N95 respirators, of which 38.2% failed the fit test [5]. They found that the duckbill-shaped type had a higher failure rate than the dome-shaped mask. However, this seemed to depend on the number of shifts, masks were worn, the hours worn, and participants donning/doffing technique. On the other hand, this is the first study to evaluate the probability for the reuse of three types of N95 respirators in a quantitative method on the fit test.

In this study, a baseline fit test was conducted to ensure that all N95 respirators could be evaluated in the same manner based on the study protocol. All participants passed fit test 1, and all participants with the cup-shaped type mask successfully completed the protocol. This might be because the cup-shaped type is more durable than other types of N95 respirators due to the mask body or rubber function.

Because of a shortage of N95 respirators caused by the COVID-19 pandemic, various reuse methods are being tried all over the world. For example, the CDC described how to use and rotate the N95 respirators at regular intervals, as in the present study [2]. Ultraviolet germicidal irradiation (UVGI), vaporous hydrogen peroxide, and moist heat were also described as decontaminating methods of N95 respirators.

However, UVGI and vaporous hydrogen peroxide need very expensive machines, which leads to limited use even in the tertiary medical center. In this regard, we simply adopted the method of storing the N95 respirators in the breathable paper bag at every regular interval.

With so many types of N95 respirators, it is a critical issue to examine which type of respirators can be fitted for each healthcare worker. In addition, it is expected that the passing rate of the fit test will be decreased in time due to deformation or body fluid/blood contamination of the mask. In fact, in our study, 14.6% of participants failed to complete the protocol. Thus, the quantitatively evaluated fit test would be required at every timing of reuse.

This study has several limitations. First, this was an observational and single-center study. Moreover, this study has only examined the limited reuse of N95 respirators but not the extended use. Secondly, we stored the reused masks in a breathable paper bag but not in a robust container.

In addition, the long-term use and durability of each respirator were not examined in the current study.

Our report shows that N95 respirators can be safely reused for a short period, which was confirmed by a quantitatively assessed fit test. UVGI of the masks with a decontamination machine could further improve the safety of reusing N95 respirators. Therefore, our results need to be confirmed in the future using cohort studies.

## Conclusions

This study shows that reusing of N95 respirators would be an allowable method in the setting of shortage of N95 respirator in the COVID-19 era.
